# Supplementary material for: Reconciling Mining with the Conservation of Cave Biodiversity: A Quantitative Baseline to Help Establish Conservation Priorities
Source: PLoS One. 2016 Dec 20;11(12):e0168348. doi: 10.1371/journal.pone.0168348 (PMC5173368; doi:10.1371/journal.pone.0168348)
Supplement: S1 Dataset — (ZIP) [file pone.0168348.s002.zip › Taxa/Serra Norte/SN_2013/N5S/Lista N5S-67.pdf]

CAVIDADE N5S-0067

Classe	Ordem	Fam/Outros	Gên/Outros	Espécie	S-U	U-S	Zon.
Arachnida	Amblypygi	Phrynidae	*Heterophrynus*	*longicornis*		1	E
Arachnida	Araneae	Araneidae		jovem	X		E
Arachnida	Araneae	Corinnidae		jovem	1		E
Arachnida	Araneae	Filistatidae		sp.1		X	E
Arachnida	Araneae	Mysmenidae	*Maymena*	sp.1		X	E
Arachnida	Araneae	Mysmenidae	*Microdipoena*	sp.1		X	E
Arachnida	Araneae	Oonopidae	aff. *Triaeris*	sp.1		X	E
Arachnida	Araneae	Pholcidae	*Leptopholcus*	sp.1		X	E
Arachnida	Araneae	Pholcidae	*Mesabolivar*	*aurantiacus*		X	E
Arachnida	Araneae	Pholcidae	*Mesabolivar*	sp.1	X		E
Arachnida	Araneae	Salticidae		jovem		X	E
Arachnida	Araneae	Scytodidae	*Scytodes*	sp.1		1	E
Arachnida	Araneae	Tetragnathidae	*Leucauge*	sp.1		X	E
Arachnida	Araneae	Theraphosidae		jovem		3	E
Arachnida	Araneae	Theridiosomatidae	*Plato*	sp.2		X	E
Arachnida	Araneae	Uloboridae		jovem	X		E
Arachnida	Pseudoscorpiones	Olpiidae		sp.1		X	E
Chilopoda	Geophilomorpha	Geophilidae		sp.2		1	E
Entognatha	Diplura	Campodeidae		sp.1		X	E
Insecta	Blattodea	Blattellidae		sp.1		1	E
Insecta	Diptera	Cecidomyiidae	Cecidomyiinae	sp.	X		E
Insecta	Diptera	Ceratopogonidae		sp.	X		E
Insecta	Diptera	Chironomidae		sp.	X		E
Insecta	Diptera	Dolichopodidae		sp.	X		E
Insecta	Diptera	Keroplatidae	*Euceroplatus*	sp.		X	E
Insecta	Diptera	Psychodidae	Phlebotominae	sp.		X	E
Insecta	Heteroptera	Lygaeidae		jovem		X	E
Insecta	Heteroptera	Reduviidae	*Zelurus*	sp.	2	2	E
Insecta	Hymenoptera	Formicidae	*Crematogaster*	sp.1	X		E
Insecta	Hymenoptera	Formicidae	*Gnamptogenys*	sp.1		X	E
Insecta	Hymenoptera	Formicidae	*Odontomachus*	sp.3	X		E
Insecta	Hymenoptera	Formicidae	*Paratrechina*	sp.1	X	X	E
Insecta	Hymenoptera	Formicidae	*Pheidole*	sp.2	X	X	E
Insecta	Isoptera	Termitidae	*Nasutitermes*	sp.	X	X	E
Insecta	Lepidoptera	Noctuoidea		sp.3		1	E
Insecta	Lepidoptera	Tineoidea		sp.1	X		E
Insecta	Lepidoptera	Tineoidea		sp.3	X		E
Insecta	Orthoptera	Phalangopsidae	*Paraclodes*	sp.		6	E
Insecta	Psocoptera	Lepidopsocidae	*Lepidopsocus*	sp.1	X		E
Insecta	Psocoptera	Psyllipsocidae	*Psyllipsocus*	sp.4		X	E
Amphibia	Anura			sp.	1		E
Mammalia	Chiroptera	Phyllostomidae	*Carollia*	*perspicillata*		3	E
Mammalia	Chiroptera	Phyllostomidae	*Diphylla*	*ecaudata*		1	E
Mammalia	Chiroptera	Phyllostomidae	*Phyllostomus*	*latifolius*		1	E
